# Di-μ-iodido-bis­[(biphenyl-2-yl)(triphenyl­phosphane-κ*P*)palladium(II)]

**DOI:** 10.1107/S1600536812047733

**Published:** 2012-11-28

**Authors:** Shayne Brown, Guy Crundwell

**Affiliations:** a Department of Chemistry, Central Connecticut State University, New Britain, CT 06053, USA

## Abstract

In the title compound, [Pd_2_(C_12_H_9_)_2_I_2_(C_18_H_15_P)_2_], the dimeric complex mol­ecule lies about an inversion center. The Pd⋯I⋯Pd bridges are slightly asymmetric, with Pd—I distances of 2.6709 (6) and 2.7486 (7) Å. The metal atom has a slightly puckered square-planar CI_2_P environment, the largest deviation from the least-squares plane being 0.143 (8) Å.

## Related literature

For crystal structures containing Pt_2_I_2_ units, see: Grushin & Alper (1993 ▶); Marshall *et al.* (2001 ▶); Lang *et al.* (2006 ▶).
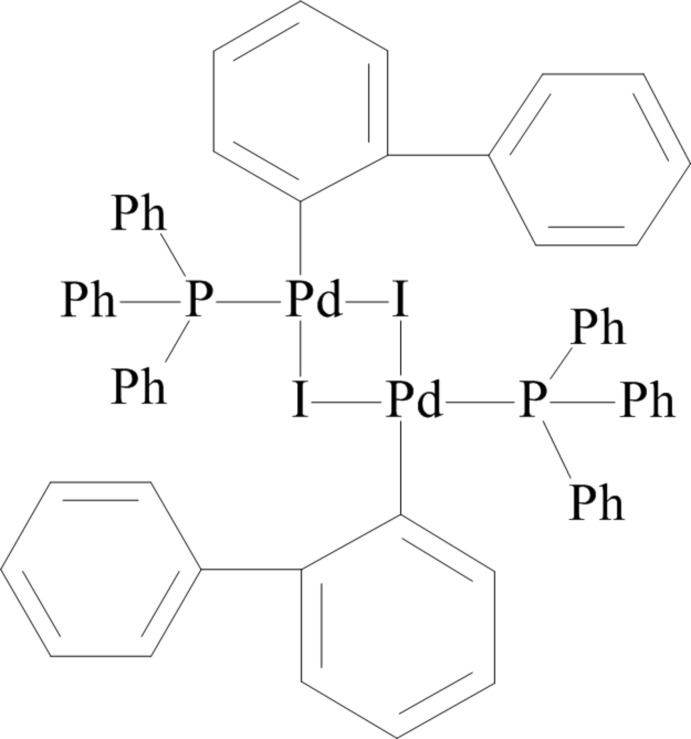


## Experimental

### 

#### Crystal data

[Pd_2_(C_12_H_9_)_2_I_2_(C_18_H_15_P)_2_]
*M* *_r_* = 1297.52
Monoclinic, 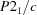

*a* = 9.6957 (4) Å
*b* = 20.0969 (10) Å
*c* = 18.3718 (7) Å
β = 133.962 (4)°
*V* = 2576.8 (2) Å^3^
*Z* = 2
Mo *K*α radiation
μ = 2.00 mm^−1^
*T* = 293 K
0.25 × 0.18 × 0.12 mm

#### Data collection

Oxford Diffraction Xcalibur Sapphire3 diffractometer
Absorption correction: multi-scan (*CrysAlis RED*; Oxford Diffraction, 2009 ▶) *T*_min_ = 0.845, *T*_max_ = 1.000
12030 measured reflections
6087 independent reflections
3783 reflections with *I* > 2σ(*I*)
*R*_int_ = 0.035

#### Refinement

*R*[*F* ^2^ > 2σ(*F* ^2^)] = 0.057
*wR*(*F* ^2^) = 0.134
*S* = 1.02
6087 reflections
298 parameters
H-atom parameters constrained
Δρ_max_ = 1.92 e Å^−3^
Δρ_min_ = −0.77 e Å^−3^

### 

Data collection: *CrysAlis CCD* (Oxford Diffraction, 2009 ▶); cell refinement: *CrysAlis PRO* (Oxford Diffraction, 2009 ▶); data reduction: *CrysAlis RED* (Oxford Diffraction, 2009 ▶); program(s) used to solve structure: *SHELXS97* (Sheldrick, 2008 ▶); program(s) used to refine structure: *SHELXL97* (Sheldrick, 2008 ▶); molecular graphics: *PLATON* (Spek, 2009 ▶); software used to prepare material for publication: *SHELXTL* (Sheldrick, 2008 ▶).

## Supplementary Material

Crystal structure: contains datablock(s) global, I. DOI: 10.1107/S1600536812047733/yk2076sup1.cif

Structure factors: contains datablock(s) I. DOI: 10.1107/S1600536812047733/yk2076Isup2.hkl

Additional supplementary materials:  crystallographic information; 3D view; checkCIF report

## Figures and Tables

**Fig. 1.**
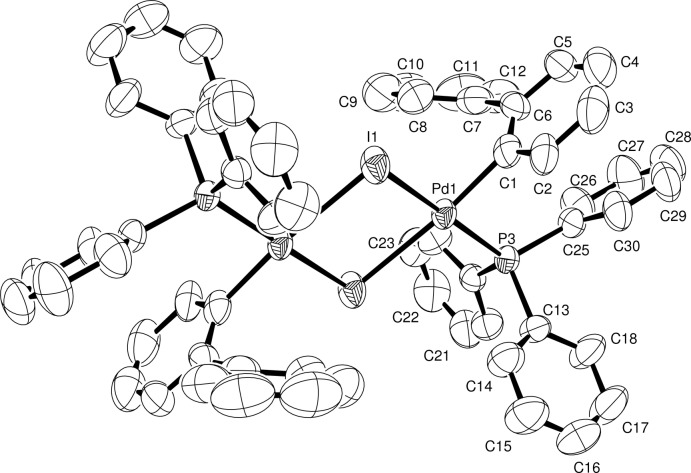
A view of the title compound. Displacement ellipsoids are drawn at the 50% probability level.

## supplementary crystallographic information

### Comment

The molecule of the title compound sits on a crystallographic inversion center; thereby requiring only half the molecule to be defined in the asymmetric unit and resulting in a *trans*- configuration. The metal centers are a slightly puckered square planar geometry. Like similar compounds containing bridging iodines, the iodines are not centered directly between the two metals; instead they have distances of 2.6709 (6) Å and 2.7486 (7) Å from the metal centers [Grushin & Alper, 1993; Marshall *et al.*, 2001; & Lang *et al.*, 2006]. The biphenyl ring is not planar; within the molecule one ring has an angle of 51.89 (39)° relative to the other. All other bond lengths and angles fall within normal values.

### Experimental

To a 100 mL three-neck round bottom flask, 0.500 grams of tetrakis(triphenylphosphane)palladium(0) (C_72_H_60_P_4_Pd, 4.33 *x* 10 ^-4^ mol) was dissolved in 20 ml anhydrous toluene under a stream of Ar gas. With stirring, 1.82 ml of 2-iodobiphenyl (C_12_H_9_I, 6.49 *x* 10 ^-4^ mol) was added. The entire reaction was stirred under Ar gas at room temperature for six days. Small yellow crystals formed on the side of the round bottom flask after four days of slow evaporation of the toluene. The crystals slowly decomposed upon heating, being completely destroyed before reaching 150°C.

### Refinement

Hydrogen atoms were included in calculated positions with a C—H distance of 0.93 Å and were included in the refinement in riding motion approximation with *U*_iso_ = 1.2*U*_eq_ of the carrier atom.

### Crystal data

**Table:**

[Pd_2_(C_12_H_9_)_2_I_2_(C_18_H_15_P)_2_]	*F*(000) = 1272
*M**_r_* = 1297.52	*D*_x_ = 1.672 Mg m^−^^3^
Monoclinic, *P*2_1_/*c*	Melting point: 423 K
Hall symbol: -P 2ybc	Mo *K*α radiation, λ = 0.71073 Å
*a* = 9.6957 (4) Å	Cell parameters from 4701 reflections
*b* = 20.0969 (10) Å	θ = 4.1–29.6°
*c* = 18.3718 (7) Å	µ = 2.00 mm^−^^1^
β = 133.962 (4)°	*T* = 293 K
*V* = 2576.8 (2) Å^3^	Block, yellow
*Z* = 2	0.25 × 0.18 × 0.12 mm

### Data collection

**Table:**

Oxford Diffraction Xcalibur Sapphire3 diffractometer	6087 independent reflections
Radiation source: fine-focus sealed tube	3783 reflections with *I* > 2σ(*I*)
Graphite monochromator	*R*_int_ = 0.035
Detector resolution: 16.1790 pixels mm^-1^	θ_max_ = 28.3°, θ_min_ = 4.3°
ω scans	*h* = −12→10
Absorption correction: multi-scan (*CrysAlis RED*; Oxford Diffraction, 2009)	*k* = −25→19
*T*_min_ = 0.845, *T*_max_ = 1.000	*l* = −24→23
12030 measured reflections	

### Refinement

**Table:**

Refinement on *F*^2^	Primary atom site location: structure-invariant direct methods
Least-squares matrix: full	Secondary atom site location: difference Fourier map
*R*[*F*^2^ > 2σ(*F*^2^)] = 0.057	Hydrogen site location: inferred from neighbouring sites
*wR*(*F*^2^) = 0.134	H-atom parameters constrained
*S* = 1.02	*w* = 1/[σ^2^(*F*_o_^2^) + (0.0678*P*)^2^ + 0.4388*P*] where *P* = (*F*_o_^2^ + 2*F*_c_^2^)/3
6087 reflections	(Δ/σ)_max_ = 0.001
298 parameters	Δρ_max_ = 1.92 e Å^−^^3^
0 restraints	Δρ_min_ = −0.77 e Å^−^^3^

### Special details

**Table:**

Experimental. Empirical absorption correction using spherical harmonics implemented in SCALE3 ABSPACK scaling algorithm (CrysAlis RED; Oxford Diffraction, 2009)Hydrogen atoms were placed in calculated positions with C—H distances of 0.93 Å and were included in the refinement in riding motion approximation with *U*_iso_ = 1.2*U*_eq_ of the carrier atom.
Geometry. All e.s.d.'s (except the e.s.d. in the dihedral angle between two l.s. planes) are estimated using the full covariance matrix. The cell e.s.d.'s are taken into account individually in the estimation of e.s.d.'s in distances, angles and torsion angles; correlations between e.s.d.'s in cell parameters are only used when they are defined by crystal symmetry. An approximate (isotropic) treatment of cell e.s.d.'s is used for estimating e.s.d.'s involving l.s. planes.
Refinement. Refinement of *F*^2^ against ALL reflections. The weighted *R*-factor *wR* and goodness of fit *S* are based on *F*^2^, conventional *R*-factors *R* are based on *F*, with *F* set to zero for negative *F*^2^. The threshold expression of *F*^2^ > σ(*F*^2^) is used only for calculating *R*-factors(gt) *etc*. and is not relevant to the choice of reflections for refinement. *R*-factors based on *F*^2^ are statistically about twice as large as those based on *F*, and *R*- factors based on ALL data will be even larger.

### Fractional atomic coordinates and isotropic or equivalent isotropic displacement parameters (Å^2^)

**Table:**

	*x*	*y*	*z*	*U*_iso_*/*U*_eq_	
Pd1	0.79025 (6)	0.05054 (2)	0.37739 (3)	0.04167 (15)	
I1	1.16659 (5)	0.06784 (2)	0.53368 (3)	0.05752 (17)	
C1	0.7754 (8)	0.1416 (4)	0.3248 (4)	0.0551 (17)	
C2	0.8449 (9)	0.1495 (4)	0.2822 (5)	0.067 (2)	
H2	0.9040	0.1142	0.2801	0.081*	
C3	0.8265 (13)	0.2129 (6)	0.2404 (6)	0.088 (3)	
H3	0.8758	0.2197	0.2121	0.106*	
C4	0.7348 (15)	0.2634 (5)	0.2430 (7)	0.100 (3)	
H4	0.7177	0.3040	0.2135	0.120*	
C5	0.6681 (12)	0.2555 (4)	0.2877 (6)	0.083 (3)	
H5	0.6088	0.2910	0.2895	0.099*	
C6	0.6881 (9)	0.1952 (3)	0.3305 (5)	0.0588 (18)	
C7	0.6180 (11)	0.1889 (4)	0.3831 (6)	0.068 (2)	
C8	0.7372 (13)	0.1662 (4)	0.4818 (6)	0.079 (2)	
H8	0.8631	0.1549	0.5171	0.095*	
C9	0.6734 (18)	0.1603 (5)	0.5279 (8)	0.106 (3)	
H9	0.7567	0.1455	0.5944	0.127*	
C10	0.489 (2)	0.1758 (6)	0.4780 (12)	0.119 (4)	
H10	0.4438	0.1685	0.5086	0.143*	
C11	0.3707 (18)	0.2021 (6)	0.3832 (11)	0.112 (4)	
H11	0.2478	0.2155	0.3512	0.134*	
C12	0.4308 (12)	0.2092 (5)	0.3339 (8)	0.092 (3)	
H12	0.3492	0.2272	0.2691	0.110*	
P3	0.4805 (2)	0.03204 (8)	0.23017 (10)	0.0410 (4)	
C13	0.4662 (8)	−0.0292 (3)	0.1506 (4)	0.0480 (15)	
C14	0.6076 (11)	−0.0734 (4)	0.1914 (6)	0.071 (2)	
H14	0.7146	−0.0723	0.2607	0.085*	
C15	0.5987 (12)	−0.1206 (4)	0.1328 (7)	0.086 (3)	
H15	0.6977	−0.1510	0.1627	0.103*	
C16	0.4449 (14)	−0.1217 (5)	0.0326 (7)	0.086 (3)	
H16	0.4369	−0.1535	−0.0071	0.104*	
C17	0.3022 (14)	−0.0773 (5)	−0.0111 (6)	0.093 (3)	
H17	0.1976	−0.0781	−0.0808	0.112*	
C18	0.3103 (11)	−0.0303 (4)	0.0472 (5)	0.080 (2)	
H18	0.2114	0.0002	0.0168	0.097*	
C19	0.3357 (8)	−0.0016 (3)	0.2508 (4)	0.0459 (14)	
C20	0.1854 (10)	−0.0459 (4)	0.1846 (5)	0.0637 (19)	
H20	0.1653	−0.0646	0.1315	0.076*	
C21	0.0680 (11)	−0.0617 (4)	0.1982 (7)	0.080 (2)	
H21	−0.0323	−0.0914	0.1542	0.096*	
C22	0.0958 (12)	−0.0342 (5)	0.2767 (7)	0.087 (3)	
H22	0.0118	−0.0442	0.2836	0.105*	
C23	0.2466 (12)	0.0077 (5)	0.3437 (7)	0.079 (2)	
H23	0.2669	0.0255	0.3973	0.095*	
C24	0.3684 (9)	0.0237 (4)	0.3323 (5)	0.0603 (18)	
H24	0.4727	0.0514	0.3789	0.072*	
C25	0.3342 (8)	0.1009 (3)	0.1431 (4)	0.0488 (15)	
C26	0.1787 (10)	0.1252 (4)	0.1242 (6)	0.075 (2)	
H26	0.1425	0.1057	0.1548	0.090*	
C27	0.0765 (12)	0.1788 (5)	0.0592 (7)	0.097 (3)	
H27	−0.0303	0.1947	0.0449	0.116*	
C28	0.1319 (12)	0.2082 (5)	0.0165 (7)	0.089 (3)	
H28	0.0675	0.2458	−0.0234	0.107*	
C29	0.2765 (12)	0.1840 (4)	0.0309 (6)	0.080 (2)	
H29	0.3082	0.2032	−0.0019	0.097*	
C30	0.3794 (10)	0.1307 (4)	0.0942 (5)	0.068 (2)	
H30	0.4813	0.1143	0.1042	0.082*	

### Atomic displacement parameters (Å^2^)

**Table:**

	*U*^11^	*U*^22^	*U*^33^	*U*^12^	*U*^13^	*U*^23^
Pd1	0.0298 (2)	0.0497 (3)	0.0306 (2)	0.00165 (18)	0.01537 (18)	0.00405 (19)
I1	0.0344 (2)	0.0661 (3)	0.0443 (2)	−0.00152 (18)	0.01692 (19)	0.0123 (2)
C1	0.035 (3)	0.065 (5)	0.035 (3)	−0.013 (3)	0.013 (3)	0.002 (3)
C2	0.053 (4)	0.087 (6)	0.041 (4)	−0.005 (4)	0.025 (3)	0.011 (4)
C3	0.081 (6)	0.118 (9)	0.057 (5)	−0.021 (5)	0.045 (5)	0.010 (5)
C4	0.102 (7)	0.092 (8)	0.064 (5)	−0.038 (6)	0.043 (6)	0.008 (5)
C5	0.088 (6)	0.052 (5)	0.061 (5)	−0.008 (4)	0.034 (5)	0.006 (4)
C6	0.053 (4)	0.042 (4)	0.049 (4)	−0.007 (3)	0.023 (3)	0.003 (3)
C7	0.071 (5)	0.050 (5)	0.074 (5)	−0.003 (4)	0.047 (5)	−0.012 (4)
C8	0.098 (6)	0.075 (6)	0.073 (5)	0.003 (5)	0.062 (5)	−0.008 (4)
C9	0.161 (11)	0.092 (8)	0.106 (8)	0.002 (7)	0.108 (9)	−0.007 (6)
C10	0.149 (12)	0.107 (10)	0.156 (12)	−0.014 (8)	0.125 (11)	−0.031 (8)
C11	0.110 (9)	0.109 (9)	0.152 (11)	−0.009 (7)	0.104 (9)	−0.040 (8)
C12	0.076 (6)	0.078 (7)	0.099 (7)	−0.003 (4)	0.053 (6)	−0.020 (5)
P3	0.0330 (7)	0.0439 (10)	0.0318 (7)	0.0029 (6)	0.0172 (6)	0.0024 (6)
C13	0.044 (3)	0.047 (4)	0.045 (3)	0.000 (3)	0.028 (3)	−0.008 (3)
C14	0.069 (5)	0.075 (6)	0.064 (5)	0.007 (4)	0.045 (4)	−0.008 (4)
C15	0.080 (6)	0.091 (7)	0.084 (6)	0.010 (5)	0.056 (5)	−0.015 (5)
C16	0.101 (7)	0.092 (7)	0.085 (6)	−0.006 (5)	0.071 (6)	−0.027 (5)
C17	0.093 (7)	0.112 (8)	0.054 (5)	−0.004 (5)	0.043 (5)	−0.023 (5)
C18	0.067 (5)	0.088 (6)	0.043 (4)	0.012 (4)	0.022 (4)	−0.015 (4)
C19	0.041 (3)	0.044 (4)	0.043 (3)	0.002 (3)	0.026 (3)	0.004 (3)
C20	0.053 (4)	0.066 (5)	0.057 (4)	−0.011 (3)	0.033 (4)	−0.004 (4)
C21	0.060 (5)	0.085 (6)	0.072 (5)	−0.022 (4)	0.037 (4)	0.006 (4)
C22	0.063 (5)	0.116 (8)	0.091 (6)	−0.013 (5)	0.056 (5)	0.008 (6)
C23	0.077 (5)	0.101 (7)	0.082 (5)	−0.001 (5)	0.064 (5)	0.001 (5)
C24	0.053 (4)	0.068 (5)	0.059 (4)	−0.010 (3)	0.038 (4)	−0.014 (4)
C25	0.032 (3)	0.051 (4)	0.037 (3)	0.001 (3)	0.014 (3)	0.003 (3)
C26	0.056 (4)	0.075 (6)	0.076 (5)	0.019 (4)	0.039 (4)	0.020 (4)
C27	0.074 (6)	0.104 (8)	0.097 (7)	0.047 (5)	0.054 (6)	0.040 (6)
C28	0.067 (5)	0.076 (6)	0.076 (6)	0.023 (4)	0.031 (5)	0.030 (5)
C29	0.072 (5)	0.075 (6)	0.056 (4)	−0.001 (4)	0.031 (4)	0.023 (4)
C30	0.053 (4)	0.077 (6)	0.053 (4)	0.007 (4)	0.028 (4)	0.020 (4)

### Geometric parameters (Å, º)

**Table:**

Pd1—C1	2.028 (7)	C14—C15	1.392 (10)
Pd1—P3	2.2792 (14)	C14—H14	0.9300
Pd1—I1	2.6709 (6)	C15—C16	1.343 (11)
Pd1—I1^i^	2.7486 (7)	C15—H15	0.9300
I1—Pd1^i^	2.7486 (7)	C16—C17	1.347 (12)
C1—C2	1.349 (9)	C16—H16	0.9300
C1—C6	1.417 (10)	C17—C18	1.389 (11)
C2—C3	1.434 (12)	C17—H17	0.9300
C2—H2	0.9300	C18—H18	0.9300
C3—C4	1.372 (13)	C19—C20	1.391 (9)
C3—H3	0.9300	C19—C24	1.392 (8)
C4—C5	1.361 (12)	C20—C21	1.362 (10)
C4—H4	0.9300	C20—H20	0.9300
C5—C6	1.385 (10)	C21—C22	1.384 (12)
C5—H5	0.9300	C21—H21	0.9300
C6—C7	1.525 (10)	C22—C23	1.366 (12)
C7—C8	1.386 (11)	C22—H22	0.9300
C7—C12	1.414 (10)	C23—C24	1.374 (9)
C8—C9	1.356 (11)	C23—H23	0.9300
C8—H8	0.9300	C24—H24	0.9300
C9—C10	1.366 (14)	C25—C26	1.380 (9)
C9—H9	0.9300	C25—C30	1.380 (9)
C10—C11	1.362 (15)	C26—C27	1.389 (11)
C10—H10	0.9300	C26—H26	0.9300
C11—C12	1.385 (13)	C27—C28	1.354 (11)
C11—H11	0.9300	C27—H27	0.9300
C12—H12	0.9300	C28—C29	1.326 (11)
P3—C19	1.821 (6)	C28—H28	0.9300
P3—C25	1.827 (6)	C29—C30	1.373 (10)
P3—C13	1.842 (6)	C29—H29	0.9300
C13—C14	1.342 (9)	C30—H30	0.9300
C13—C18	1.381 (9)		
			
C1—Pd1—P3	89.00 (16)	C13—C14—C15	121.9 (8)
C1—Pd1—I1	89.25 (16)	C13—C14—H14	119.1
P3—Pd1—I1	171.22 (4)	C15—C14—H14	119.1
C1—Pd1—I1^i^	174.70 (18)	C16—C15—C14	119.1 (8)
P3—Pd1—I1^i^	95.49 (4)	C16—C15—H15	120.5
I1—Pd1—I1^i^	86.730 (18)	C14—C15—H15	120.5
Pd1—I1—Pd1^i^	93.270 (18)	C15—C16—C17	120.6 (8)
C2—C1—C6	121.2 (7)	C15—C16—H16	119.7
C2—C1—Pd1	118.9 (6)	C17—C16—H16	119.7
C6—C1—Pd1	119.9 (5)	C16—C17—C18	120.4 (8)
C1—C2—C3	119.2 (8)	C16—C17—H17	119.8
C1—C2—H2	120.4	C18—C17—H17	119.8
C3—C2—H2	120.4	C13—C18—C17	119.6 (7)
C4—C3—C2	118.7 (8)	C13—C18—H18	120.2
C4—C3—H3	120.6	C17—C18—H18	120.2
C2—C3—H3	120.6	C20—C19—C24	119.5 (6)
C5—C4—C3	121.8 (9)	C20—C19—P3	123.1 (5)
C5—C4—H4	119.1	C24—C19—P3	117.1 (5)
C3—C4—H4	119.1	C21—C20—C19	119.3 (7)
C4—C5—C6	120.4 (9)	C21—C20—H20	120.3
C4—C5—H5	119.8	C19—C20—H20	120.3
C6—C5—H5	119.8	C20—C21—C22	121.0 (7)
C5—C6—C1	118.6 (7)	C20—C21—H21	119.5
C5—C6—C7	119.0 (7)	C22—C21—H21	119.5
C1—C6—C7	122.4 (6)	C23—C22—C21	119.9 (8)
C8—C7—C12	117.8 (8)	C23—C22—H22	120.1
C8—C7—C6	121.5 (7)	C21—C22—H22	120.1
C12—C7—C6	120.6 (8)	C22—C23—C24	120.1 (8)
C9—C8—C7	121.2 (9)	C22—C23—H23	120.0
C9—C8—H8	119.4	C24—C23—H23	120.0
C7—C8—H8	119.4	C23—C24—C19	120.1 (7)
C8—C9—C10	121.0 (11)	C23—C24—H24	120.0
C8—C9—H9	119.5	C19—C24—H24	120.0
C10—C9—H9	119.5	C26—C25—C30	118.0 (6)
C11—C10—C9	119.5 (12)	C26—C25—P3	122.5 (5)
C11—C10—H10	120.3	C30—C25—P3	119.6 (5)
C9—C10—H10	120.3	C25—C26—C27	119.5 (8)
C10—C11—C12	121.1 (11)	C25—C26—H26	120.2
C10—C11—H11	119.5	C27—C26—H26	120.2
C12—C11—H11	119.5	C28—C27—C26	120.2 (8)
C11—C12—C7	119.2 (10)	C28—C27—H27	119.9
C11—C12—H12	120.4	C26—C27—H27	119.9
C7—C12—H12	120.4	C29—C28—C27	121.0 (8)
C19—P3—C25	102.8 (3)	C29—C28—H28	119.5
C19—P3—C13	105.9 (3)	C27—C28—H28	119.5
C25—P3—C13	103.1 (3)	C28—C29—C30	120.1 (8)
C19—P3—Pd1	112.63 (19)	C28—C29—H29	119.9
C25—P3—Pd1	119.9 (2)	C30—C29—H29	119.9
C13—P3—Pd1	111.23 (19)	C29—C30—C25	121.1 (7)
C14—C13—C18	118.5 (7)	C29—C30—H30	119.5
C14—C13—P3	120.9 (5)	C25—C30—H30	119.5
C18—C13—P3	120.6 (5)		

Symmetry code: (i) −*x*+2, −*y*, −*z*+1.
